# Ureteral duplicity with a left ureterocele: a case report

**DOI:** 10.11604/pamj.2024.47.21.39242

**Published:** 2024-01-18

**Authors:** Reda Tariqi, Hamza EL Abidi, Imad Boualaoui, Ahmed Ibrahimi, Hachem El Sayegh, Yassine Nouini

**Affiliations:** 1 Department of Urologic Surgery “A”, Ibn Sina University Hospital, Mohammed V University, Rabat, Morocco

**Keywords:** Ureteral duplicity, ureterocele, endoscopic ureterocelotomy, case report

## Abstract

An intravesical ureterocele is a rare condition in which a terminal ureter terminates in a cystic dilation of the bladder. We present the case of a 42-year-old female who presented with irritative lower urinary tract symptoms and left lower back pain. Computed tomography (CT) urography revealed ureteral duplication with a ureterocele complicated by upper tract obstruction. Treatment involved endoscopic ureterocelotomy, which successfully relieved symptoms and resolved renal obstruction.

## Introduction

Ureteral duplications are relatively uncommon, found in 0.9% of routine autopsies and 0.3% of uroscans [1]. According to the Weigert-Meyer law, the upper ureter usually opens medially and the lower ureter usually opens laterally. Intact ureteral duplication may be associated with other congenital anomalies such as short intramural ureters or vesicoureteral reflux or reflux with upper ureteral occlusion leading to obstruction.

Ureteroceles, characterized by distal ureteral dilation in the bladder, are primarily a pediatric condition. However, we report an unusual case of ureteral duplicity with a left ureterocele in an adult, emphasizing the importance of considering ureteral anomalies in adult patients with urologic symptoms [2,3].

## Patient and observation

**Patient information:** the patient is a 42-year-old female with a history of uterine fibroid surgery in 2014. She presented with irritative lower urinary tract symptoms and left lumbago persisting for several years.

**Clinical findings:** on clinical examination, the patient appeared generally well, and apyretic, with urinalysis indicating bacterial presence and leukocytes. There was no lumbar sensitivity, and the external genital examination was unremarkable.

**Timeline of the current episode:** the patient's symptoms had been evolving for several years, leading to the current presentation.

**Figure 1:**
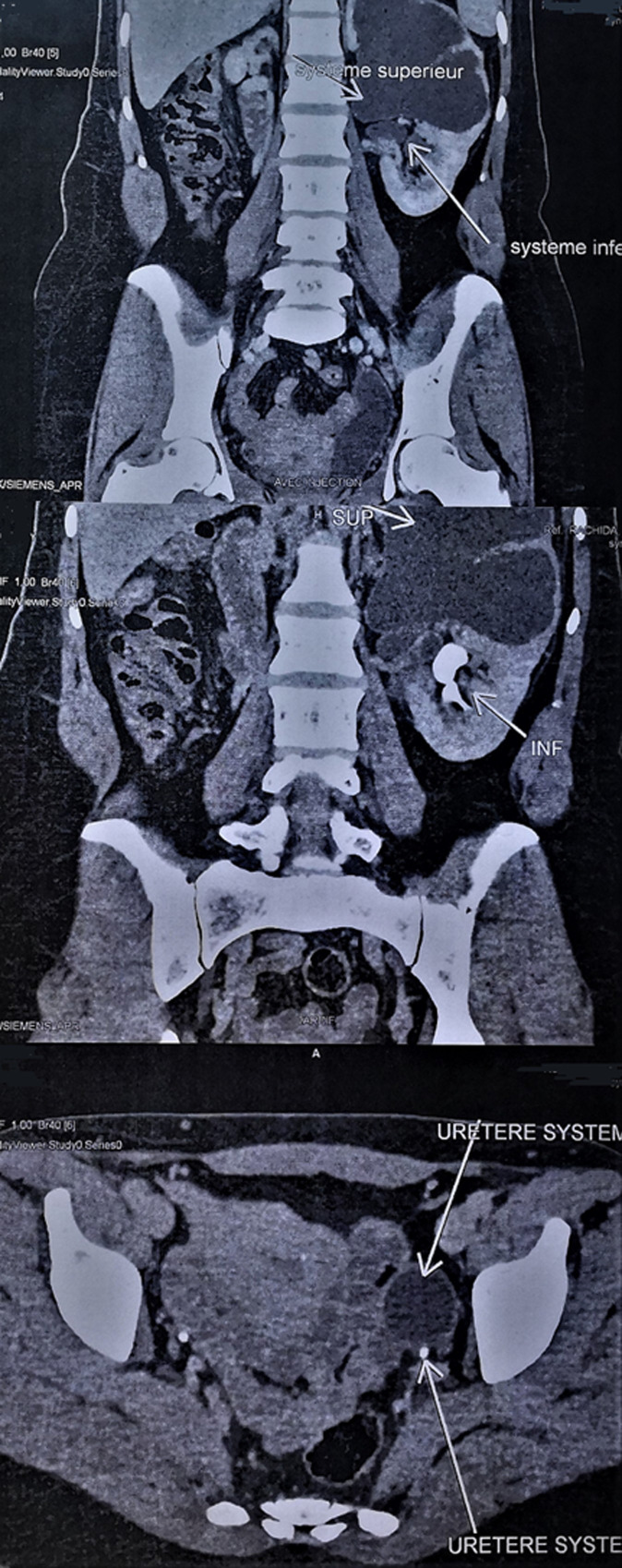
uroscan image of ureterocele and double renal system; computed tomography uroscan showed an enlarged left kidney measuring 169.5 mm in height and a double system with an ureterocele and ureterohydronephrosis of the upper system with a renal pelvis measuring 41.8 mm in anteroposterior diameter, a laminated aspect of the renal parenchyma and excretory delay

**Diagnostic assessment:** initial renal ultrasound revealed a double pyelocaliceal system in the left kidney, with dilatation of the upper system (31 mm) causing thinning of the renal parenchyma and dilatation of the lower system (18 mm) with preserved cortico-sinusal differentiation of the renal parenchyma. A 16-slice spiral CT urogram with intravenous contrast showed an enlarged left kidney (169.5 mm) with a double system and a ureterocele and ureterohydronephrosis of the upper system with a renal pelvis measuring 41.8 mm in the anteroposterior diameter, a laminated aspect of the renal parenchyma and excretory delay. The lower system is not dilated, secreting and excreting within the physiological time frame of a normal ureteral outlet (Figure 1).

**Diagnosis:** the patient was diagnosed with ureteral duplicity and a left ureterocele complicated by upper tract obstruction.

**Figure 2:**
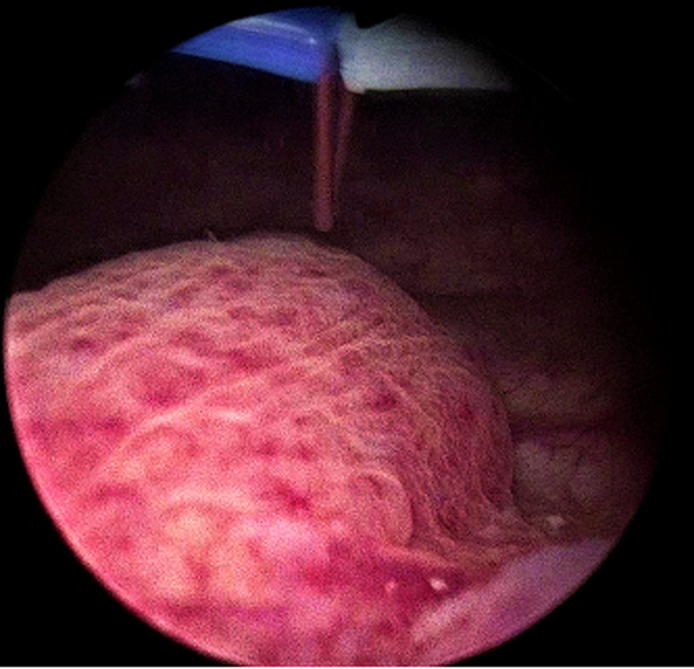
cystoscopic image of the ureterocele; ureterocele on the left lateral bladder wall, crossing the midline

**Figure 3:**
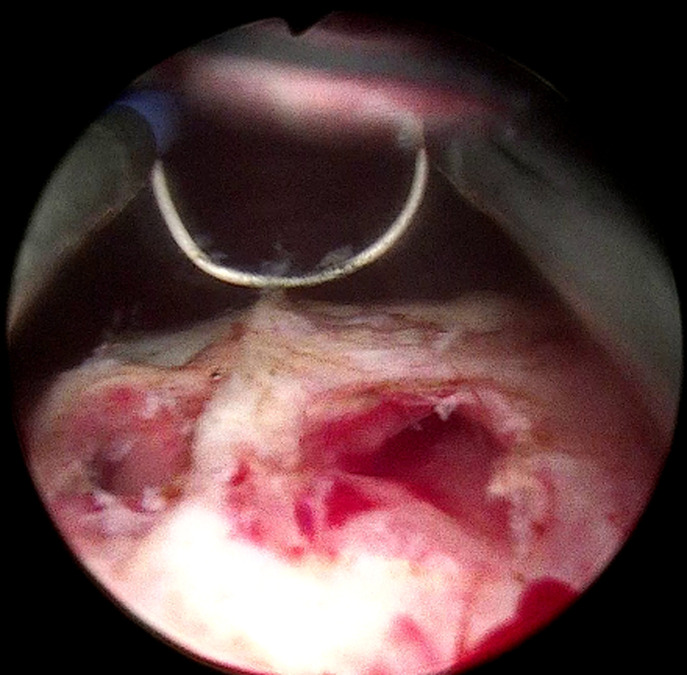
cystoscopic image after ureterocelotomy; ureterocelotomy was performed using puncture-electrocoagulation

**Therapeutic interventions:** under spinal anesthesia, the patient underwent endoscopic ureterocelotomy. A 30° rigid cystoscope was used to visualize the ureterocele on the left lateral bladder wall, crossing the midline (Figure 2). Ureterocelotomy was performed using puncture-electrocoagulation (Figure 3).

**Follow-up and outcome of interventions:** postoperative follow-up included clinical examination, ultrasound, and renal diethylene triamine penta-acetic acid (DTPA) scintigraphy performed three months after ureterocele treatment, showing improvement in drainage and symptom resolution.

**Patient perspective:** the patient was satisfied with symptom improvement and pleased with endoscopic surgery due to its non-scarring nature.

**Informed consent:** the patient's consent was voluntary and informed.

## Discussion

Ureteroceles, typically diagnosed during prenatal ultrasounds or early childhood evaluations, are a relatively rare occurrence. However, there are exceptional cases where individuals may manifest ureterocele-related symptoms later in life [4]. The imperative for treatment stems from the risk of chronic kidney damage due to obstructive uropathy. Ureteral duplication, often associated with ureteroceles, is primarily attributed to the premature division of ureteral buds, remnants of the Wolffian duct, or the presence of two separate ureteral buds [5]. It is believed to follow an autosomal dominant inheritance pattern with incomplete penetrance [6]. Remarkably, this condition tends to be more prevalent in Caucasian females, mirroring the demographic characteristics of our case [7].

The clinical presentation of ureteral duplication can vary considerably based on the patient's age [5]. In many instances, patients with double ureters remain asymptomatic, with diagnosis typically occurring incidentally. However, symptoms may manifest differently, with recurrent urinary tract infections and vesicourethral reflux often observed in infancy, while adults may experience symptoms such as low back pain and obstructive complications [8].

A retrospective study involving 26 adult patients demonstrated favorable outcomes following transurethral incisions of the ureterocele [9]. This approach proved to be safe and effective, although some cases exhibited mild vesicoureteral reflux even after intervention. Given the diverse symptomatology associated with ureteroceles, imaging studies, including renal bladder ultrasonography and CT urography, play pivotal roles in diagnosing and preventing permanent complications resulting from chronic obstruction.

Based on insights from existing literature and clinical trends, the initial treatment strategy for patients requiring surgical intervention for ureteroceles, regardless of whether they have simplex or duplex systems, should typically involve transurethral cystoscopic incision. Reintervention, while reserved for clinically significant events, may not be necessary for many patients, thereby obviating the need for more invasive procedures. In most instances, the non-functioning upper pole can be safely retained. Addressing associated vesicourethral reflux should be considered only if it proves clinically significant [10].

## Conclusion

Ureteral duplicity with a ureterocele is a rare condition, more commonly seen in pediatric patients. This case underscores the importance of considering ureteral anomalies in adults with urologic symptoms. Endoscopic ureterocelotomy is an effective treatment option for this condition, offering symptom relief and improved renal function.
